# Exploring Small Extracellular Vesicles for Precision Medicine in Prostate Cancer

**DOI:** 10.3389/fonc.2018.00221

**Published:** 2018-06-13

**Authors:** Matteo Giulietti, Matteo Santoni, Alessia Cimadamore, Francesco Carrozza, Francesco Piva, Liang Cheng, Antonio Lopez-Beltran, Marina Scarpelli, Nicola Battelli, Rodolfo Montironi

**Affiliations:** ^1^Department of Specialistic Clinical and Odontostomatological Sciences, Polytechnic University of Marche, Ancona, Italy; ^2^Oncology Unit, Macerata Hospital, Macerata, Italy; ^3^Section of Pathological Anatomy, School of Medicine, United Hospitals, Polytechnic University of the Marche Region, Ancona, Italy; ^4^Oncology Unit, City Hospital, Faenza, Italy; ^5^Department of Pathology and Laboratory Medicine, Indiana University School of Medicine, Indianapolis, IN, United States; ^6^Department of Surgery, Cordoba University Medical School, Cordoba, Spain

**Keywords:** prostate cancer, drug resistance, tumor microenvironment, tumor biomarkers, small extracellular vesicles

## Abstract

Tumor microenvironment constitutes a complex network in which tumor cells communicate among them and with stromal and immune cells. It has been shown that cancer cells are able to exchange genetic materials through small extracellular vesicles (EVs), a heterogeneous group of vesicles with different size and shape, cargo content, and function. The importance to investigate populations of circulating EVs would be of great importance as prostate cancer (PCa) biomarkers. In several neoplasms as well as in PCa, nanometer-sized EVs of endosomal origin are implicated in supporting tumor growth and metastatic spread by both altering local stroma cells and creating a protumor environment that favors the formation of pre-metastatic niches. Several techniques are applicable for the isolation and analysis of PCa-derived small EVs and are illustrated in this article. Due to the high sensitivity and specificity of these techniques, small EVs have become ideal candidates for early diagnosis. Moreover, we discuss the role of small EVs during PCa carcinogenesis, as well as in modulating the development of drug resistance to hormonal therapy and chemotherapy, thus underlining the potential of EV-tailored strategies in PCa patients.

## Introduction

Tumor microenvironment components represent promising therapeutic targets in cancer. Indeed, it is implicated in tumor carcinogenesis and progression, as well as in the suppression of antitumor immune response and in the development of drug resistance (1). These activities are the result of a complex network between normal and cancer cells, which supports tumor proliferation and metastatic spread. Recently, it has been shown that cancer cells are able to exchange genetic materials through extracellular vesicles (EVs), a heterogeneous group of vesicles with different size and shape, cargo content, and function (2).

Among them, nanometer-sized EVs of endosomal origin are involved in promoting tumor growth and metastasis (3) through the alteration of local stroma cells (4) and the creation of a protumor environment that sustains the formation of pre-metastatic niches (5, 6). These small size vesicles of ~30–150 nm in diameter are released into the extracellular space after fusion of multivesicular bodies with the cell membrane and could be found in blood, urine, and other biological fluids. Urine EVs have been shown to contain RNA, DNA, and miRNA, lipid and proteins/transporters specific to cells of the kidney and urogenital tract. Thus, urine represents a potentially useful non-invasive material for diagnosis and prognosis of genitourinary tract neoplasms (7, 8). Due to their involvement in the early phases of tumor development and dissemination, future diagnostic and therapeutic EV-tailored strategies are emerging in the armamentarium of cancer patients.

In genitourinary tumors, EVs represent potential novel biomarkers. Indeed, small EVs have been involved in kidney diseases and cancer, as well as in bladder cancer growth and progression (9). In prostate cancer (PCa), small EVs have been reported to contain high levels of molecules that stimulate cancer cell invasion, such as integrins β4 (ITGB4) and αvβ6, vinculin (VCL), transmembrane glycoprotein Trop-2, vimentin, and N-cadherin, well-known epithelial–mesenchymal transition markers (6, 10).

Furthermore, the efficacy of urine assays in PCa diagnostic could be improved by simple procedures like prostate massage to increase the amount of tumor-specific nucleic acids released (11). This review illustrates the main techniques for isolation and analysis of small EVs and emphasizes their role in the carcinogenesis, progression, and drug response in patients with prostate tumor, thus underlying the potential of emerging EV-tailored approaches in this setting.

## Isolation of PCa Small EVs

The currently adopted methods for small EVs isolation include differential and density gradient ultracentrifugation, size-based methods (ultrafiltration and size-exclusion chromatography), polymer-based precipitation, and immunoaffinity capture. The choice of the isolation technique depends on the sample source, the sample amount, the type of the downstream molecular analyses, the desired purity level, and final concentration. Each method shows both advantages and disadvantages, summarized in Table 1. It should be noted that some isolation methods can be more easily integrated with analysis of vesicular RNAs and proteins, so fewer steps are needed. This can therefore improve the reliability and efficiency of small EVs analysis.

**Table 1:** Overview of various small extracellular vesicles (EVs) isolation techniques.

Isolation method	Principle	Processing time	Advantages	Disadvantages	Purity degree	Reference
Ultracentrifugation	Sedimentation rates (depending on size and shape)	3–16 h	Easy to use, high yields	High equipment cost, time-consuming, low portability, not scalable, high-speed centrifugation may induce vesicle disruption	Low: contamination by cell debris, large vesicles, vesicle aggregates, and protein complexes	(12–16)

Density gradient separation	Density	20–24 h	Adequate grade of purity	Laborious and time-consuming, low portability, not scalable	Medium: contamination by high-density lipoprotein and other vesicles	(17–20)

Ultrafiltration	Size	1–6 h	Fast, good portability, useful for urine concentration	High-speed centrifugation may induce vesicle disruption, membrane filter clogging, small EVs loss, not scalable	Low: protein co-isolation	(12, 21)

Size-exclusion chromatography	Size	6–12 h	Gravity flow preserves small EVs integrity, high reproducibility	Not scalable, time-consuming	High	(22)

Small EVs precipitation	Polymer mixtures that alter the solubility of small EVs	30 min–2 h	Fast, easy to use, no specialized equipment required, large and scalable sample capacity	Low purity, not recommended if coupled with mass spectrometry	Low: contamination by protein aggregates and other vesicles	(23–25)

Immunoaffinity capture	Presence of small EVs’ surface antigens	16–20 h	Unique method for the isolation of specific small EVs	High reagent cost, low capacity, low yields, time-consuming	Very high	(26–29)

### Ultracentrifugation

Ultracentrifugation is the most frequently used approach for small EVs isolation, mainly since it does not require high level of technical experience. This technique is based on the different sedimentation rates (depending on size and density) of small EVs from the other sample components (12). Generally, before ultracentrifugation, some steps of low-speed centrifugation (3,000–20,000 × *g*) are carried out to clean the sample from large particles, including cells, platelets, apoptotic bodies, and microvesicles. Typically, for ultracentrifugation, the used force ranges from 100,000 to 200,000 × *g*. Due to the high-speed centrifugation, the small EVs rupture represents a high risk and leads to the yield reduction of intact particles suitable for downstream analyses. It also requires high volumes of samples, it is time-consuming and not suitable for large number of samples (13).

Moreover, pellet obtained by this method may contain also EV aggregates and protein complexes similar to small EVs in size (14). A particular case regards the uromodulin (THP), which is the most abundant protein in urine and can form a polymeric network that facilitates small EVs’ aggregation. Uromodulin removal and the consequent increase of small EVs’ yield can be achieved by using the detergent CHAPS or the reducing agent dithiothreitol in urine samples (15). Finally, unlike free RNA that is degraded by urinary RNAses, DNA contamination is frequent, and a DNAse should be used if small EVs’ DNA is the target of downstream analyses (16).

### Density Gradient Centrifugation

The density gradient centrifugation method allows small EVs separation from other sample components by exploiting their typical density, which ranges between 1.13 and 1.19 g/ml (17). By using pre-constructed columns composed of solutions with different densities, after ultracentrifugation small EVs will be at a specific column layer.

It allows purer small EVs isolation than simple ultracentrifugation; in fact, it is generally used as a second step after other isolation methods to purify the extracts (18, 19). However, high-density lipoproteins represent a contaminant frequently co-isolated with small EVs; therefore, plasma and serum are not suitable sample types for density gradient centrifugation. In addition, it cannot distinguish small EVs from other EVs with similar density (20).

### Ultrafiltration

Ultrafiltration represents an efficient alternative to ultracentrifugation. It involves the use of nanomembrane filters of polyethersulfone or polyvinylidene difluoride with an approximately 50–100 kDa molecular mass cutoff (12). Generally, some steps of filtration with membrane filters having larger pores can be performed for the depletion of floating cells and large cell debris (21). Membranes allow the concentration of very diluted samples and therefore they are especially suited for cell culture media and urine samples.

Extracellular vesicles size can be also exploited by the size-exclusion chromatography that, using sepharose packed columns, allows the separation of the small EVs fraction from biofluids, with high purity and reproducibility (22). However, both ultrafiltration and size-exclusion chromatography have long run times that limit their scalability for clinical routine laboratory applications.

### Precipitation Methods

Some isolation kits for small EVs precipitation are commercially available (23, 24). They are based on water-excluding polymer mixtures that force insoluble components out. When these polymers are added to the sample and after a low-speed ultracentrifugation (<20,000 × *g*), they allow the precipitation of small EVs. Since precipitation method is very fast, easy to use, and no equipment is necessary, it is a method scalable for large sample sizes and therefore is suited for the clinical use.

Recently, performances of ultracentrifugation and four different precipitation methods have been assessed in urine samples. In this comparison, a precipitation method outperforms the others in detecting alterations of PCa small EVs’ markers (25). However, other EVs are frequently co-isolated, making this procedure not so specific for small EVs. Moreover, abundant proteins, including uromodulin, can also be recovered.

### Immunoaffinity Isolation Methods

Finally, immunoaffinity isolation methods exploit the presence of specific proteins in the small EVs’ surface (26). Antibodies conjugated with magnetic beads or other materials can recognize the vesicular antigens and facilitate the precipitation by low-speed centrifugation or the isolation by magnetic techniques. This method is highly specific, allowing the isolation of only small EVs by using general small EV surface markers (e.g., CD81, CD9, and CD63) and even only small EV subpopulations, for example, tumor small EVs by using a tumor-specific marker.

Recently, specific methods for immuno-based small EVs isolation and detection for PCa have been proposed, both measuring total small EVs (27) and exploiting the anti-prostate-specific membrane antigen (PSMA) antibody (28). Moreover, it has been observed that immunoaffinity-based methods have better performance than purification methods in isolating PCa small EVs from patient plasma samples (29). However, this generally leads to low yields. In addition, immunoaffinity isolation requires several elaboration steps making it subjected to potential errors by operators.

## Analysis of PCa Small EVs

After isolation, small EVs quantity, size, purity, and other properties can be assessed. A popular method to quantify total small EVs is the nanoparticle tracking analysis (NTA) (12, 30–32). It also allows the determination of a size distribution profile of particles in solution and, by verifying that the observed distribution ranges within typical small EV size, also the purity degree can be estimated. In particular, a laser beam makes particles visible and therefore their concentration can be assessed. The size distribution can be evaluated by measuring the velocity of the Brownian motion of each particle and, according to the Stokes–Einstein equation, speed of particles can be correlated to the particle size. However, protein complexes, lipoprotein particles, and vesicle aggregates similar to small EVs in size can be revealed by NTA and therefore the small EVs concentration could be overestimated. Flow cytometry is another popular method for both small EVs quantification and characterization (33). In particular, particles are illuminated by a laser beam, and the scattered light is detected. However, currently, there is a detection limit of 150 nm; therefore, the use of latex or magnetic beads coupling with small EVs is suggested to make these aggregates visible by flow cytometry (34). Flow cytometry has been used to perform “liquid biopsy” in PCa patient plasma samples. Biggs et al. measured circulating prostate microparticles (PMPs), a type of EVs, with a size ranging from 100 to 1,000 nm, immunoreactive to anti-PSMA mAb. PMP enumeration levels in plasma permit to identify patients with Gleason score ≥8 PCa independently from PSA value (35).

Regarding small EVs quantification, researchers frequently adopt the enzyme-linked immunosorbent assay (ELISA). Similar to immunoaffinity isolation methods, this approach exploits small EV surface antigens that can be detected by specific antibodies. To quantify total small EVs, CD63, CD9, and CD81 vesicular-specific proteins are generally measured by ELISA (34). The evaluation of the enzymatic activity of the acetyl-CoA acetylcholinesterase, which is enriched in small EVs, is another method used for quantification and purity assessments. Alternatively, to evaluate the purity degree of a small EVs preparation, Western blotting can be used to assess the presence of typical small vesicular proteins (CD63, CD9, CD81, Alix, and Tsg101) (12). However, this assay is not quantitative and gives no information on the particle size.

Upon small EVs isolation and quantification, their RNA, DNA, protein, and lipid content can be analyzed. RNA profiling can include mRNAs and miRNAs and can be achieved both by PCR-based assays for single RNA molecule profiling and by next-generation sequencing (RNA-seq) for large-scale analysis. For example, after isolation of small EVs in urine specimens, the levels of PCa-associated mRNA transcripts in these vesicles have been assessed by quantitative real-time PCR analysis (36, 37). In addition, quantification of vesicular miRNAs can be assessed by RNA-seq, as carried out for urinary small EVs from PCa patients (38). However, vesicular miRNA quantification can be affected by inadequate procedural choices, including the choice of optimal endogenous miRNA for expression normalization (39). Differential expression analysis of vesicular mRNAs or miRNAs between samples from primary PCa, metastatic cancers, and healthy subjects can lead to the identification of candidate biomarkers (40). In addition, other gene expression analysis methods, including co-expression network analysis, can suggest potential diagnostic and prognostic biomarkers (41–43). Analysis of urinary vesicular RNA and DNA has also been performed in prostate tumors to identify the presence of mutations (44). It has been observed that small EVs released by different PCa cell lines harbor specific DNA mutations (45). These results may allow the development of non-invasive tests for mutational status assessment in PCa. Interestingly, the specific molecular effects due to the identified mutations on transcription, splicing, miRNA binding at 3′UTR, and nucleocytoplasmic export of mRNA can be effectively predicted (46–49).

Furthermore, methods for single protein and multiple protein profiling exist. Western blot, cytofluorimetry, and ELISA represent popular methods for the assessment of specific small vesicular proteins and for validation of results obtained by other proteomic methods, also regarding urinary small EVs of PCa patients (50). Recently, a new aptamer-based method for the simultaneous analysis of around 1,000 proteins from small EVs of PCa patients has been developed (51). Moreover, mass spectrometry has been frequently used to identify all small EVs’ proteins from many sample types and, recently, it has also been exploited for the analysis of small EVs’ proteins in plasma samples of PCa patients (52). Interestingly, mass spectrometry can be also used to assess the lipid content of small EVs. In particular, lipid species in small EVs released by PCa cell lines and present in urine of PCa patients have been evaluated (53, 54). It should be also taken into account the heterogeneous nature of cancer EVs; different types of PCa cells are more prone to shed heterogeneous populations of EVs compared with other PCa cells. Indeed, El-Sayed and her group have recently showed mesenchymal-like prostate carcinoma cells have a propensity to release EVs of varied sizes with diameters ranging from 100 to 300 nm approximately, while epithelial-like PCa cells principally generate small vesicles (50–150 nm). The isolation method could be a technical limitation; further studies are needed to properly characterize and classify these vesicles and to investigate the potential effects of EVs as a whole, including small EVs’ fraction and others EVs’ subgroups (10).

## Exploring Small EVs in PCa Patients

### Small EVs and PCa Diagnosis

In the past 10 years, small EVs have emerged as a novel effective and non-invasive clinical tool for the screening and early diagnosis of PCa. This evidence is supported by the specificity and sensitivity showed by small EVs analysis in this disease, together with the possibility of a non-invasive assessment of gene expression and mutations (36), thus leading to a year-by-year growing number of studies in this context. Indeed, it has been shown that only PCa patients are characterized by high levels of nanovesicles (125–180 nm, i.e., small EVs) expressing both CD81 and PSA (55). Such results were obtained analyzing 1 ml of plasma samples of 15 healthy donors, 15 benign prostatic hyperplasia, and 15 PCa patients by nanoscale flow cytometry and ELISA assay. Moreover, McKiernan and his group revealed that gene expression assay in urine small EVs was able to discriminate high-grade (Gleason score ≥7) from low-grade (GS6) cancer and benign disease. Small EVs’ RNA cycle threshold values of ERG, PCA3, and SPDEF were used to derive urine small EVs gene expression assay score that was tested in 255 men and then was prospectively validated in an independent cohort of 519 men. In this way, they identified patients with higher-grade PCa among men with elevated PSA levels, thus potentially reducing the number of unnecessary biopsies (56). In the same view, high Claudin 3 levels from isolated small EVs were able to predict GS ≥8 (57), while small EVs’ levels of gamma-glutamyltransferase 1, a cell-surface enzyme that regulates the catabolism of extracellular glutathione, were significantly higher in PCa patients compared with benign prostatic hyperplasia patients (58).

Interestingly, several miRNAs from isolated urinary small EVs, such as miR-2909 (59), miR-19b (60), miRNA-21, and miR-375 (61), have demonstrated to be effective as diagnostic biomarkers for prostate tumors. The isolation method used in these studies included precipitation of small EVs in fluids with low-speed centrifugation step (59) or differential centrifugation (60, 61) with selection of vesicles of 30–100 nm obtained by 0.1-µm filtration (60). The authenticity of these isolated small EVs was confirmed by subjecting them to morphological analysis by electronic microscopy (59, 61), immunostaining with antibodies against CD63, CD9, and CD24 (60), or Western blot analysis using CD63 antibody coupled with standard immunodetection procedure (59).

More recently, the analysis of the lipidome of urinary small EVs showed statistically significant difference between PCa patients (*n* = 15) and healthy volunteers (*n* = 13), in particular for the high presence of phosphatidylserine and lactosylceramide associated with PCa diagnosis (53). These pioneering studies, even if limited to a small number of samples, are promising and should be validated in larger independent cohorts.

### Small EVs and PCa Development and Progression

Prostate cancer-derived small EVs are directly involved in PCa carcinogenesis and metastasis. Indeed, small EVs are able to significantly reduce apoptosis, increase cancer cell proliferation, and induce cell migration in LNCaP and RWPE-1 cells (62). Moreover, they can modulate bone cell formation by affecting the fusion and differentiation of osteoclasts in the metastatic sites (63), thus favoring the formation of pre-metastatic niches.

Hypoxia plays a crucial role in regulating small EVs activity. In fact, it has been shown that small EVs secreted by PCa cells under hypoxic conditions showed higher metalloproteinases (MMPs) activity and increased levels of proteins primarily implicated in the remodeling of epithelial adherens junction pathway compared with small EVs released from normoxic cells. Therefore, this enhanced the invasiveness and stemness of naïve PCa cells (64).

Beyond their potential use for PCa diagnosis, miRNAs are directly implicated in PCa development and progression. It has been shown that normal prostate fibroblasts (WPMY-1) transfected with miR-100-5p, miR-21-5p, and miR-139-5p augmented their migration and metastatic invasion by increasing the expression of MMP-2, -9, and -13 and RANKL (65). Furthermore, adipocyte differentiation-related protein can be detected in small EVs released by PCa cells and is able to induce neuroendocrine differentiation of these cells in a paracrine fashion (66). Interestingly, small EVs are also involved in modulating PCa-induced immunosuppression of dendritic cell functions (67) and promote immune evasion by downregulating NKG2D expression on natural killer cells and CD8+ T cells (68).

Even if most of the studies on biological effects of small EVs are conducted *in vitro*, they lay the foundations for following clinical investigations.

Recently, it has been shown that the expression of CD9 is increased in PCa of patients who suffer from disease recurrence in 5 years, indicating the role of CD9 in the progression of recurrent advanced PCa (69).

Using the established immunocapture and immunodetection method, Soekmadji and colleagues reported that the level of CD9+ EVs in plasma is increased in PCa patients compared to those with benign prostate hyperplasia and, on the contrary, CD63+ EVs level does not show a significant difference between the two groups. Moreover, they showed that in plasma obtained from a metastatic PCa patient cohort the level of CD9+ EVs were higher in circulating tumor cell (CTC)-positive PCa patients compared with CTC-negative patients. Instead, the CD63+ EVs level did not show significant differences between the cohorts (70, 71).

Such findings underline the importance to investigate particularly subpopulations of circulating EVs that would be more informative as PCa biomarkers.

### Therapeutic Potential of Small EVs and Drug Resistance in Patients With PCa

The stability and low immunogenicity of EVs support their use as therapeutic delivery agents for cancer drugs and small molecules. The methodology for loading EVs with a therapeutic cargo consists into two different approaches: the first one is based on the indirect modification of EV membranes *via* the genetic engineering of their parental cell (72). The second one needs the direct encapsulation of a cargo into purified exosomes through active (i.e., sonification and electroporation) or passive (i.e., the introduction of hydrophobic drugs into EVs, the utilization of multivalent electrostatic interactions, permeabilization with saponin) loading methods (72). These technologies will represent a major step forward in the era of precision medicine for PCa patients and should be tested into future clinical trials.

Small EVs have been shown to be crucial for the development of drug resistance in patients with prostate tumor. In 2017, Del Re et al. assessed AR-V7 as a predictor of resistance to hormonal therapy by highly sensitive digital droplet polymerase chain reaction in plasma-derived small EVs’ RNA. They found that both median progression-free survival (20 vs. 3 months; *p* < 0.001) and overall survival (8 months vs. not reached; *p* < 0.001) were significantly longer in AR-V7-negative vs. AR-V7-positive patients (73).

Small EV-derived microRNAs contribute also to PCa chemoresistance (74) and can act as surrogate biomarker of tumor response to taxanes (75). It has been observed that the transfer of small EVs (in particular, small EVs’ MDR-1/P-gp) from docetaxel-resistant cell lines to DU145, 22Rv1, and LNCap PCa cell lines induces acquired resistance to this drug (76). In the same view, Kawakami et al. reported that β4 (ITGB4) and VCL in small EVs could be useful markers of PCa progression correlated with taxane resistance (77).

Interestingly, serum small EVs’ P-glycoprotein high levels are associated with resistance to docetaxel but not to cabazitaxel (78), thus representing a potential biomarker to guide the decision-making process in PCa patients.

Another important therapeutic use of EVs is vaccination to treat cancer. It has already been studied on mice injected with autologous tumor-derived nanovesicles, enriched with tumor-specific antigens. The high-level immunogenicity of these nanovesicles induced an antitumor immune responses in both primary and metastatic melanoma mouse models (79).

Some questions have to be answered before these preclinical evidences on EV-based therapy can be applied in a clinical scenario. Major problems are the EV’s production, purification, and concentration, the biodistribution of EVs, the targeting of recipient cells, the up-taking by the recipient cells, and the effects on the recipient immune system (80).

## Discussion and Conclusion

For years, Gleason score has represented the most relevant prognostic factor in patients with PCa, along with PSA levels measured at the time of diagnosis and the TNM score. Plasma quantification of PSA has markedly improved the early detection of PCa, but still lacks the required specificity. More recently, a new generation of potentially predictive and prognostic parameters has grown, opening the way to molecularly tailored approaches in PCa patients. Small EVs have been reported to act as mediators of cell-to-cell communication due to the regulatory functions of their content. The high sensitivity and specificity of data obtained from small EVs analysis, their presence in almost all human fluids, and the variety of their functions exerted during tumor carcinogenesis, progression, and response to treatments, support the notion that small EVs exploration will represent a cornerstone of future approaches in cancer patients. Further investigations are needed, particularly regarding the content of the different EVs and their possible use in clinical and therapeutic settings.

The current application of EVs engineering is limited by a series of key factors that should be overcrossed to introduce these methodologies into daily clinical practice. Among them, a standardization of present methods of isolation and analysis results fundamental. Interestingly, EVs is a wide term that includes different vesicle types such as exosomes, microvesicles, and apoptotic bodies. This nomenclature, based on size, biogenesis, and cellular release mechanisms, is currently not consistent throughout the literature (17, 81, 82). Although the International Society of EVs (ISEV) is making an effort to unify this controversial nomenclature, favoring the term EVs instead of exosomes and of other terms, a consensus criterion does not still exist (82). ISEV is involved also in the standardization of the methodologies of EVs isolation and characterization (81). Also in this case, there are not commonly accepted procedures. The lack of a consensus regarding EVs isolation and even storing conditions (83) can result in impure exosome preparations, raising a possible explanation regarding some inconsistent results in literature.

**Figure 1:**
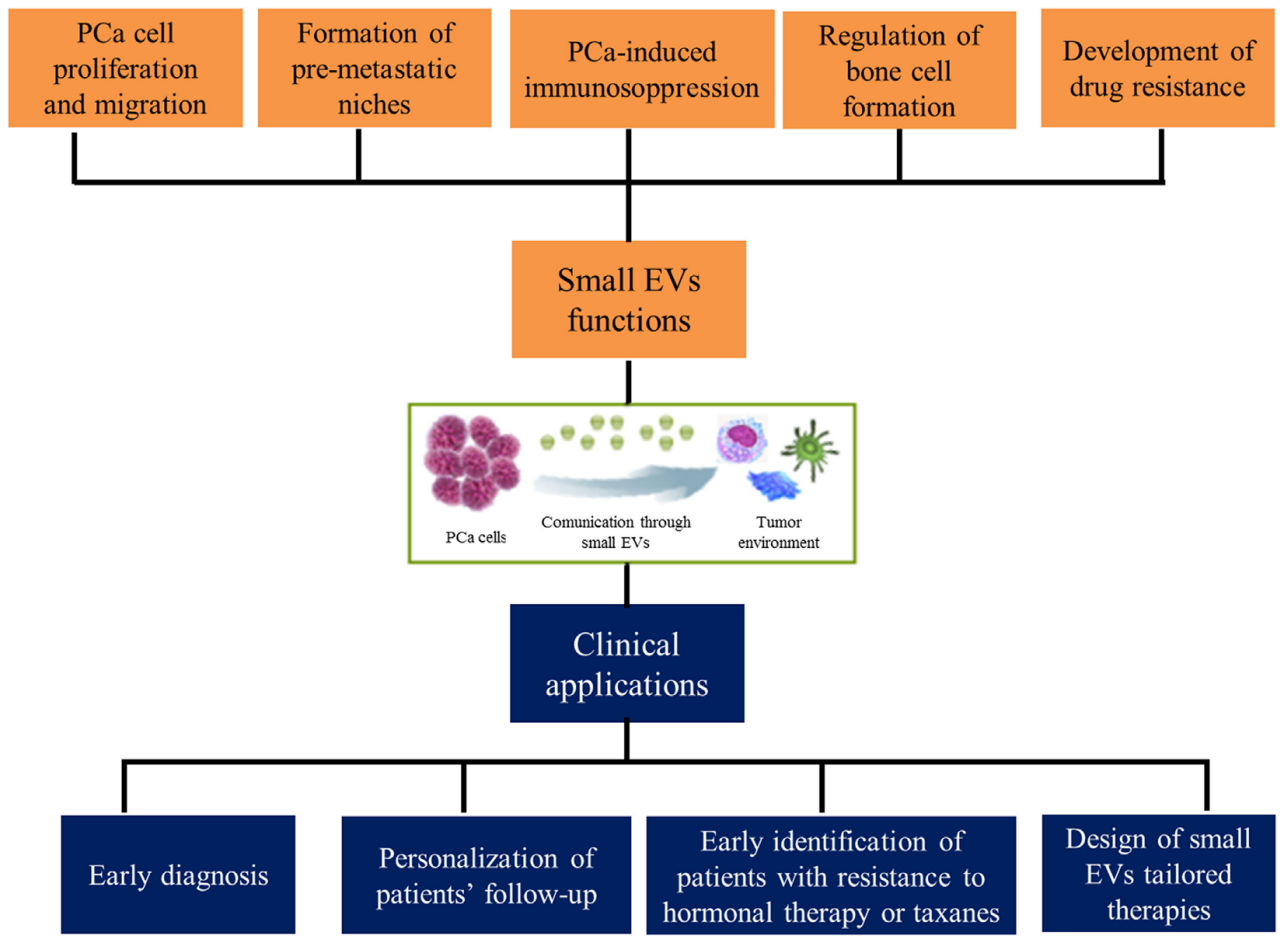
Functions exerted by small extracellular vesicles (EVs) in prostate cancer (PCa) microenvironment and their clinical implications.

In conclusion, the search for effective predictive and prognostic factors in prostate neoplasms is still ongoing. The growing knowledge about molecular biology of this tumor is bringing us very rapidly to a new age of therapeutic possibilities. Nevertheless, the cost–benefit ratio of the massive application of these new potential prognostic factors represents a crucial point in the choice of the “best one.” Based on this scenario, small EVs may represent a cornerstone in the future diagnostic and treatment-decision processes of PCa, leading to a more tailored and personalized approach for patients with advanced disease (Figure 1) (84).

## Author Contributions

RM and MS (Marina Scarpelli): conception and design. MG and MS (Matteo Santoni): drafting the manuscript. FC, FP, and AC: review of the literature. LC, NB, and AL-B: critical revision of the manuscript.

## Conflict of Interest Statement

The authors declare that the research was conducted in the absence of any commercial or financial relationships that could be construed as a potential conflict of interest.
